# Patient-Reported Symptom Severity in a Nationwide Myasthenia Gravis Cohort: Cross-sectional Analysis of the Swedish GEMG Study

**DOI:** 10.1212/WNL.0000000000012604

**Published:** 2021-10-05

**Authors:** Malin Petersson, Amalia Feresiadou, Daniel Jons, Andreea Ilinca, Fredrik Lundin, Rune Johansson, Anna Budzianowska, Anna-Karin Roos, Viktor Kågström, Martin Gunnarsson, Peter Sundström, Fredrik Piehl, Susanna Brauner

**Affiliations:** From the Department of Clinical Neuroscience (M.P., F.P., S.B.), Karolinska Institutet, Stockholm; Department of Neuroscience, Neurology (A.F.), Uppsala University; Department of Clinical Neuroscience, Institute of Neuroscience and Physiology (D.J.), the Sahlgrenska Academy, University of Gothenburg; Department of Neurology (D.J.), Sahlgrenska University Hospital, Gothenburg; Department of Clinical Sciences Lund, Neurology (A.I.), Skåne University Hospital, Lund University, Malmö; Departments of Neurology (F.L.) and Biomedical and Clinical Sciences (F.L., A.B.), Division of Neurobiology, Linköping University; Department of Neurology and Rehabilitation (R.J.), Karlstad Central Hospital; Department of Internal Medicine in Jönköping (A.B.), Section of Neurology, Region Jönköping County; Department of Clinical Science, Neurosciences (A.-K.R.), Unit of Neurology, Umeå University, Östersund; Rehabilitation Clinic (V.K.), Sundsvall Hospital; Department of Neurology, Faculty of Medicine and Health (M.G.), Örebro University; Department of Clinical Science, Neurosciences (P.S.), Umeå University; and Department of Neurology (F.P., S.B.), Karolinska University Hospital, Stockholm, Sweden.

## Abstract

**Background and Objectives:**

To describe myasthenia gravis activities of daily living (MG-ADL) in relation to clinical characteristics in a large Swedish nationwide cohort.

**Methods:**

In a cross-sectional prevalence cohort study, the Genes and Environment in Myasthenia Gravis study, performed from November 2018 through August 2019, patients with myasthenia gravis (MG) were invited to submit an extensive 106-item life environment questionnaire, including the MG-ADL score. Patients were classified into early-onset MG (EOMG, <50 years), late-onset MG (LOMG, ≥50 years), or thymoma-associated MG (TAMG). Comparisons of disease-specific characteristics were made between subgroups, sexes, and different MG-ADL scores.

**Results:**

A total of 1,077 patients were included, yielding a 74% response rate: 505 (47%) were classified as EOMG, 520 (48%) LOMG, and 45 (4%) TAMG. Mean age at inclusion was 64.3 years (SD 15.7) and mean disease duration was 14.6 years (SD 14.0). Complete MG-ADL scores (n = 1,035) ranged from 0p to 18p, where 26% reported a score of 0p. Higher MG-ADL scores were associated with female sex, obesity, and diagnostic delay (odds ratio [OR] 1.62, 1.72, and 1.69; *p*_adj_ = 0.017, 0.013, and 0.008) and inversely correlated with high educational attainment (OR 0.59; *p*_adj_ = 0.02), but not with age at inclusion, disease subtype, or disease duration. Almost half of the population (47%) reported MG-ADL ≥3p, corresponding to an unsatisfactory symptom state.

**Discussion:**

In this nationwide study, comprising more than 40% of the prevalent MG population in Sweden, almost half of the patients reported current disease symptoms associated with an unsatisfactory symptom state, indicating the need for improved treatment options.

Myasthenia gravis (MG) is an autoantibody-mediated neuroinflammatory disease characterized by fluctuating muscle weakness and muscular fatigability.^1,2^ Clinical management has evolved considerably during the past century,^3^ but MG still causes serious morbidity and a substantial effect on patients' quality of life.^4^ Patient-reported outcome measures (PROMs) are gaining increasing attention, both for MG and other chronic inflammatory diseases.^5^ MG-specific PROMs, such as MG activities of daily living (MG-ADL),^6^ more readily reflect the disease activity over time compared to point-in-time evaluations,^7^ which is particularly important in a disease like MG, given its known fluctuation during the day. MG-ADL correlates well to several other MG outcome measures such as the quantitative myasthenia gravis score, MG composite, as well as the MG 15-item Quality of Life Scale.^7,8^ PROMs are estimated by the patient independently and have been increasingly used in clinical trials.^9-12^

The prevalence of MG in Sweden was estimated to be 24.8/100,000 in 2010.^13^ Prevalence has increased dramatically in the past century. The incidence has also increased in past decades, especially in the late-onset group.^3,14-18^ The Genes and Environment in Myasthenia Gravis (GEMG) study was launched in order to provide improved information on causative factors and disease characteristics in a large nationwide cohort.

The main objective of the present study was to describe the basic and disease-specific characteristics of the GEMG study cohort in relation to symptom severity as reflected by the MG-ADL scale.

## Methods

### Study Population

**Figure 1:**
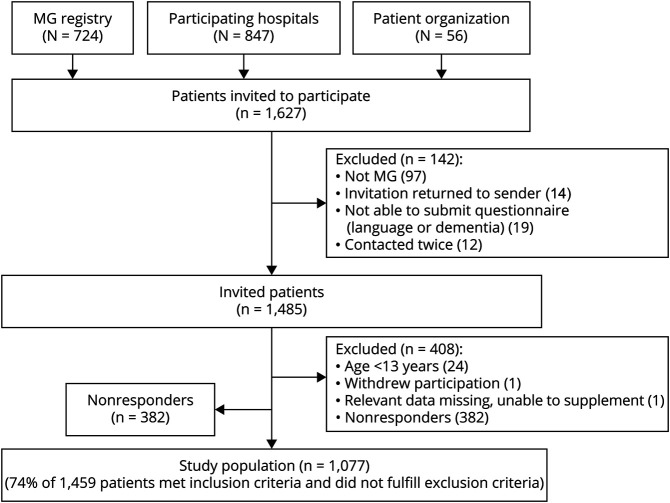
Recruitment Flowchart GEMG = Genes and Environment in Myasthenia Gravis study; MG = myasthenia gravis.

The GEMG study is a Swedish nationwide cross-sectional study of patients with MG. We invited all patients registered in the national clinical MG registry (MGreg [n = 724]; neuroreg.org), patients who contacted the study upon learning about it from the patient organization (n = 56), and all additional patients who had received the ICD code for MG (G70.0) upon clinical visits on at least 2 occasions during 2010 through 2018 at 12 collaborating hospitals including all Swedish university hospitals (n = 847) (Figure 1). MGreg was initiated in 2011 as a nationwide publicly funded quality registry to collect information on disease course and long-term outcomes with MG prospectively but remains a voluntary option for patients and participating centers. Of the 56 patients who contacted the study through the patient organization, 39 (70%) were later identified via collaborating hospitals. A total of 1,627 invitations were sent out. All patients with MG diagnosis aged 18 years or older at study start were included. Exclusion criteria were insufficient Swedish reading proficiency or disease onset <13 years of age, to avoid inclusion of patients with congenital myasthenic syndrome. All residents in Sweden have a unique personal identification number composed of their date of birth and 4 additional digits. Participating patients were identified using the personal identification number and duplicates of patients who were contacted twice by mistake were removed (n = 12). Recruitment and data collection were carried out from November 2018 to July 2019. Supplementary telephone interviews were made after reviewing all questionnaires and data lock was on August 31, 2019.

### Data Collection

Patients were invited to participate by responding to an extensive and standardized online or paper questionnaire regarding historic and present environmental and lifestyle exposures as well as disease-specific questions.^19-21^ Patients were asked to participate by responding to an online or paper questionnaire. Patients fulfilling inclusion criteria were contacted up to 4 times.

Paper questionnaires were received from 452 patients, 624 patients filled in the web-based version, and 1 patient was interviewed by telephone due to severe visual impairment. Patients submitting a paper questionnaire were older (69.3 [SD 14.1] vs 60.7 [SD 15.8] years; *p* < 0.001) and more likely to have late-onset MG (LOMG) subtype (57% vs 46%; *p* < 0.001).

Paper questionnaires were registered manually into the same database as the web-based responses, providing the full data set for further analyses.

### Outcome Variables

Patients reporting ever having a thymoma were classified into thymoma-associated MG (TAMG) and remaining patients were stratified by age at disease onset: <50 years (early-onset MG [EOMG]) and ≥50 years (LOMG). Thirty-two patients did not remember the year of onset. Of these, 25 were classified based on year of onset or diagnosis reported in MGreg (n = 10), anamnestic information on decade (n = 1), or approximate year of diagnosis (n = 14); the remaining 7 patients were not subgrouped.

Patients were asked to report symptoms at onset, at peak of disease, and at study inclusion. A patient was deemed as having generalized disease if ever reporting generalized symptoms at any of the above mentioned timepoints.

All participants were asked to report an itemized MG-ADL score, which was subsequently used to estimate patient acceptable symptom state (PASS).^9^ PASS is a 1-question-based score that has been validated in multiple chronic inflammatory diseases including MG.^22,23^ Patients respond to the question “Considering all the ways you are affected by myasthenia, if you had to stay in your current state for the next months, would you say that your current disease state status is satisfactory?” The cutoff for a satisfactory state in MG has previously been reported at 2p in MG-ADL.^9^ We therefore used this cutoff to make an estimated PASS (ePASS). An unsatisfactory symptom state, MG-ADL ≥3p, is termed negative ePASS, and a satisfactory symptom state, MG-ADL 0p–2p, is termed positive ePASS.

### Statistics

All statistical analyses were performed using R version 4.0.3^24^ and RStudio version 1.3.1093. Statistical significance was defined as *p* value <0.05. Means and SD were calculated for continuous variables and counts and frequencies for categorical variables. Differences in continuous variables were assessed by Wilcoxon rank sum test, categorical variables with all groups of n ≥ 5 were assessed by χ^2^ test, and Fisher exact test was used for categorical variables with any group of n < 5. Adjusted *p* values and odds ratios (ORs) or β values with 95% confidence interval (CI) were obtained by multivariate regression models with the dependent variable as continuous MG-ADL or MG-ADL dichotomized into 0p–5p (approximation of no severe generalized disease) or ≥6p with at least 1p in nonocular items (approximation of severe generalized disease). Patients with 6p in only ocular items (n = 5) were put into the 0p–5p category. Categorical independent explanatory variables were sex (female or male), subgroup (EOMG, LOMG, or TAMG), diagnostic delay ≥2 years (yes or no), current tobacco use (yes or no), current coffee consumption (yes or no), body mass index (<25, overweight or obese), and university degree (yes or no). Continuous independent explanatory variables were disease duration in years from onset and age at inclusion in years. Model fit statistics were used to calculate Akaike information criterion (AIC) values for both models; linear model (AIC = 4,997) and multivariate regression model (AIC = 898) and data from the multivariate model are therefore presented. Due to the exploratory nature of the study, no correction for multiple testing was performed.

### Quality Control

Internal validation of the manual registration of data from paper questionnaires was performed by one of the authors (M.P.) in order to assess potential bias and errors at data entry. Forty-nine randomly chosen paper questionnaires (approximately 10%) were reimputed into the web module. Interrater agreement was determined for each variable by calculating the Cohen kappa. The mean Cohen kappa for all variables was 0.93. Values >0.80 are considered strong.^25^

External validation was performed to assess potential recall bias. The magnitude of recall bias was estimated by comparing patient-reported data on time of disease onset, diagnosis, and thymectomy against data recorded in MGreg. Mean deviation (patient reported – physician reported), SDs, and intraclass coefficient (ICC) assessing 1-way consistency among data were calculated. The mean deviation was <1 year for disease onset, diagnosis, and thymectomy. ICCs were 0.93, 0.96, and 0.97, respectively. ICC values of 0.75–0.90 indicate good reliability and values >0.90 excellent reliability.^26^ Potential association between disease duration and difference in self-reported data and data in MGreg was also investigated by calculating the Spearman correlation coefficient for disease onset (*R* = 0.27, *p* = 1.3e-07), diagnosis (*R* = 0.29, *p* = 1e-08), and thymectomy (*R* = 0.26, *p* = 0.0084).

To further assure the representability of the cohort, a nonresponder analysis was carried out by comparing age, sex, and region of residence for responders and nonresponders.

### Standard Protocol Approvals, Registrations, and Patient Consents

The study was approved by the Regional Ethics Committee Stockholm, Sweden (2018/1,436-31) and all participating patients gave written informed consent. The study was conducted in accordance with the Strengthening the Reporting of Observational Studies in Epidemiology (STROBE) guidelines.

### Data Availability

Deidentified data relating to the study are available from the corresponding author upon reasonable request and ethics approval.

## Results

The final study population comprised 1,077 individuals of 1,459 invited eligible participants, resulting in a 74% response rate (Figure 1). We estimate that the cohort covers 42% of all patients with MG in Sweden, based on a recent prevalence estimate obtained through compulsory national health registries and the population size as of December 31, 2019.^13,27^ Nonresponders were younger (61.4 years [SD 18.3]) compared to responders (63.8 years [SD 16.0]) (*p* = 0.040). Internal and external validation assessing data entry and comparing patient to physician-reported data using MGreg displayed very strong agreement, indicating high accuracy of the dataset. A potential, but weak, correlation between disease duration and magnitude of difference in patient and physician-reported data was observed.

**Figure 2:**
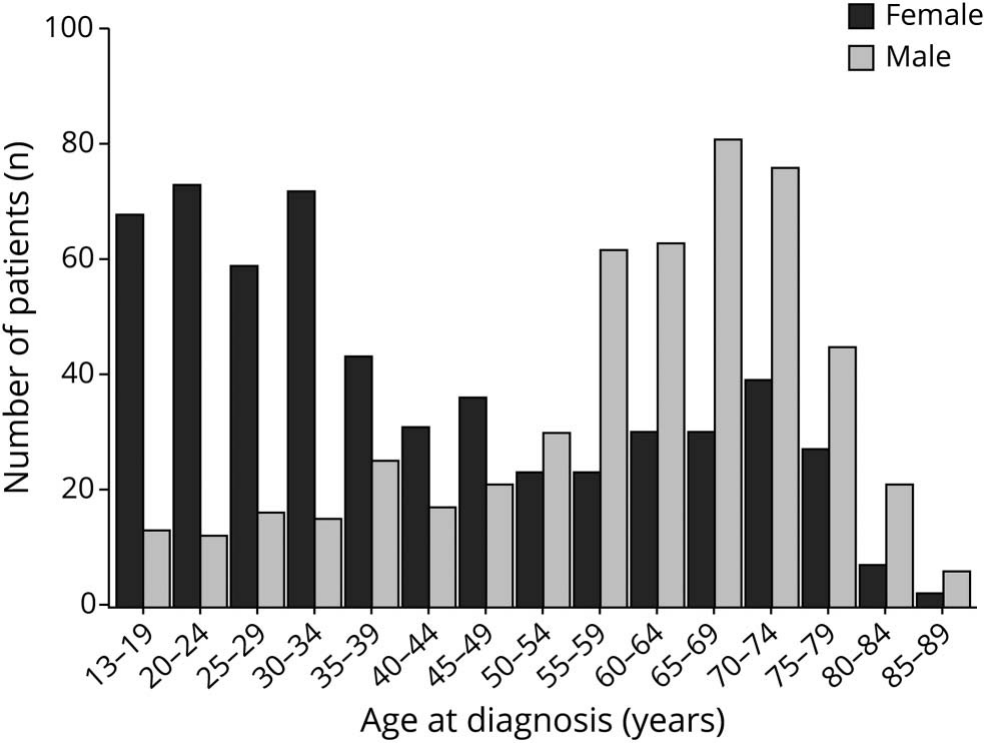
Age and Sex Age at diagnosis stratified by sex for all patients who reported a year of diagnosis (n = 1,066).

Mean age at diagnosis was 49.6 years (SD 20.1) and at inclusion 64.3 years (SD 15.7); 53% of respondents were female (Table 1). Forty-five patients (4%) reported a thymoma and were classified as TAMG and the remaining as EOMG (n = 505) or LOMG (n = 520). Age at diagnosis stratified by sex revealed a bimodal incidence peak in female patients, whereas male patients were predominantly diagnosed at 70 years of age (Figure 2). The EOMG group was dominated by female patients (76%), contrasting with the LOMG group (30%; *p* < 0.001). As expected, patients with EOMG had undergone thymectomy to a higher extent (74%) compared to LOMG (19%; *p* < 0.001). Patients with EOMG more often reported generalized symptoms at onset (50%), and LOMG, ocular symptoms (55%) (*p* < 0.001). At study inclusion, 30% reported no symptoms, and frequencies were similar in all subgroups (Table 1). Sex-stratified analyses revealed significant differences in the EOMG subgroup (Table 2). Female patients with EOMG reported onset at a significantly earlier age (27.6 years [SD 9.4]) compared to men with EOMG (33.4 [SD 10.8]; *p* < 0.001). Significantly more women with EOMG reported generalized symptoms at onset (55% vs 37%; *p* < 0.001) or ever (92% vs 74% in men with EOMG; *p* < 0.001).

**Table 1:**
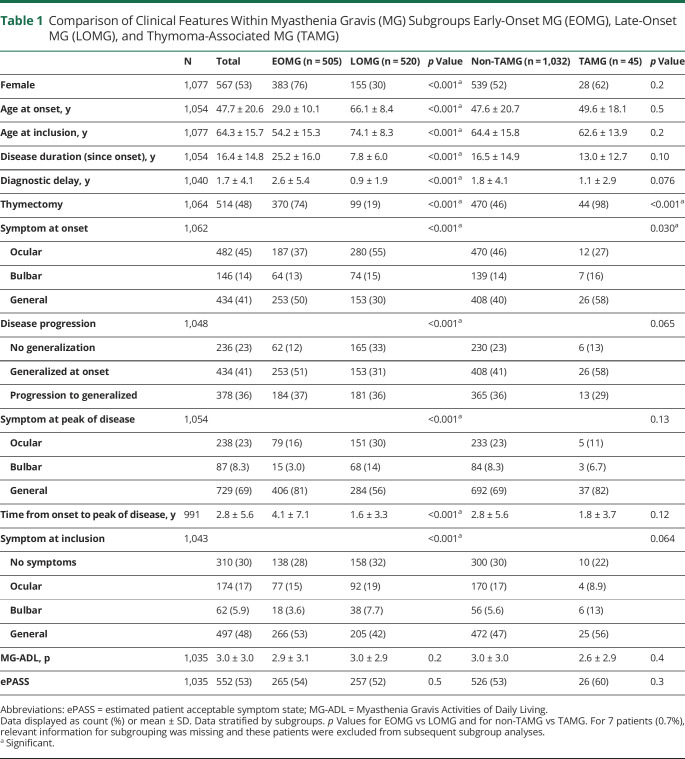
Comparison of Clinical Features Within Myasthenia Gravis (MG) Subgroups Early-Onset MG (EOMG), Late-Onset MG (LOMG), and Thymoma-Associated MG (TAMG)

**Table 2:**
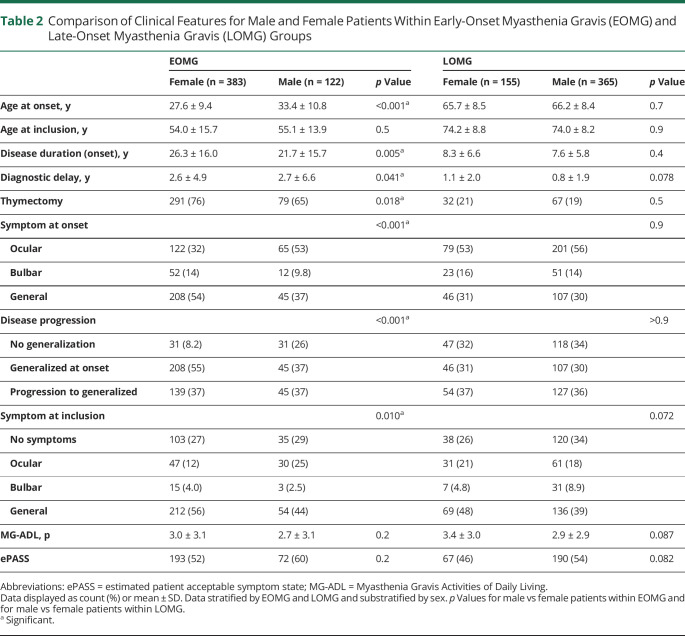
Comparison of Clinical Features for Male and Female Patients Within Early-Onset Myasthenia Gravis (EOMG) and Late-Onset Myasthenia Gravis (LOMG) Groups

**Figure 3:**
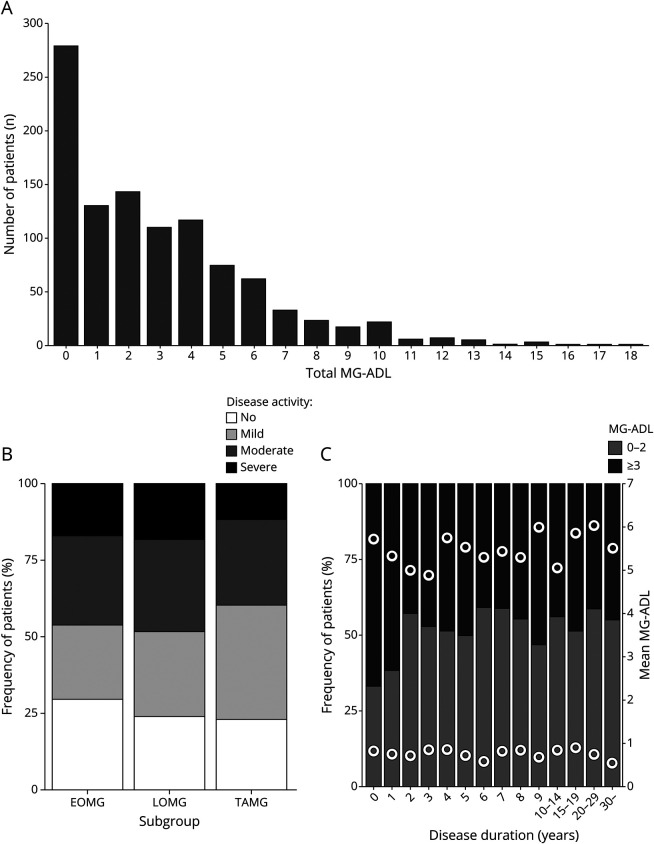
Measures of Disease Activity by Myasthenia Gravis Activities of Daily Living (MG-ADL) (A) Total MG-ADL for all patients submitting complete MG-ADL scores (n = 1,035). (B) Distribution of MG-ADL score divided into no (0p), mild (1p–2p), and moderate disease activity (3p–5p and patients with 6p limited to ocular items) as well as severe generalized disease (≥6p of which at least 1p is in nonocular items) per subgroup. (C) Distribution of estimated patient acceptable symptom state (PASS) determined by an MG-ADL cutoff at 0p–2p and ≥3p by disease duration. Mean MG-ADL within each category is displayed as filled dots. Data shown for all patients with complete MG-ADL scores and disease duration (n = 1,026). EOMG = early-onset myasthenia gravis; LOMG = late-onset myasthenia gravis; TAMG = thymoma-associated myasthenia gravis.

Disease activity was assessed by the MG-ADL scale^6^ and scores ranged between 0p and 18p (Figure 3A, Table 1). A large proportion reported 0p (26%) and similar fractions of patients with no (0p), mild (1p–2p), or moderate disease activity (3p–5p) or severe generalized disease (≥6p) were observed between the subgroups (Figure 3B). Using a multivariate regression model, comparing patients with severe generalized disease to those without minimal manifestations, we sought to identify factors correlating with higher MG-ADL score (Table 3). We observed that female sex (OR 1.62; 1.09–2.41; *p*_*adj*_ = 0.017), obesity (OR 1.72; 1.12–2.64; *p*_*adj*_ = 0.013), and diagnostic delay ≥2 years (OR 1.69; 1.14–2.48; *p*_*adj*_ = 0.008) significantly correlated with high disease activity. Having obtained a university degree (OR 0.59; 0.37–0.91; *p*_*adj*_ = 0.02) was inversely correlated with severe generalized disease. These correlations were also significant in a linear model including all patients (data not shown). In a subanalysis assessing the effect of thymectomy, neither thymectomy nor time to thymectomy was associated with MG-ADL, even when restricting the analyses to patients with EOMG (data not shown; *p*_*adj*_ > 0.1).

**Table 3:**
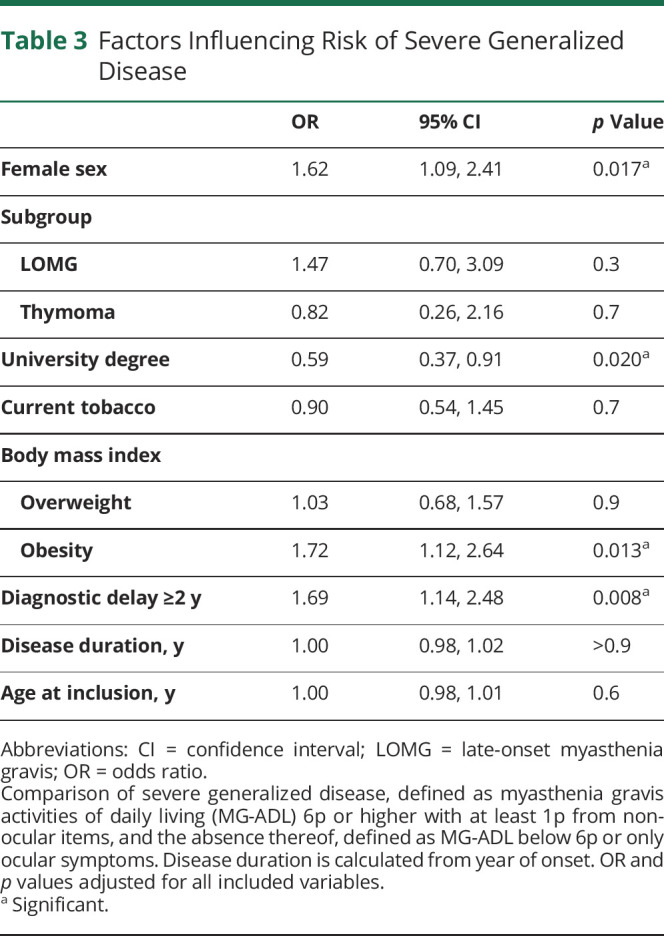
Factors Influencing Risk of Severe Generalized Disease

We thereafter derived ePASS, a measure of patient-acceptable disease burden based on patient-reported MG-ADL (Table 1). MG-ADL scores of ≥3p (i.e., ePASS negative) were reported by 47%, indicating dissatisfaction with their current disease status.^9^ Patients with MG-ADL ≥3p at study inclusion more often had general symptoms at onset compared to patients with MG-ADL 0p–2p (44% vs 37%; *p =* 0.019) and more reported ever having generalized symptoms (83% vs 73%; *p <* 0.001). Among patients who were diagnosed within 2 years prior to study inclusion, fewer reported MG-ADL 0p–2p (37%), compared to patients diagnosed earlier (55%; *p <* 0.001) (Figure 3C). This difference remained significant after adjustment for subgroup and age (OR 2.17; 1.40–3.41; *p*_*adj*_ < 0.001).

## Discussion

We report disease characteristics and patient-reported symptom state in a large nationwide prevalence MG cohort. The study had 74% response rate and is estimated to include 42% of all prevalent MG cases in Sweden, based on an epidemiologic study using compulsory national health registry data.^13^ Our major findings include a detailed characterization of disease activity based on MG-ADL, where increased score associated with female sex and overweight and inversely correlated to university education. We also estimate that, using an MG-ADL–based cutoff, almost half of the study population has an unsatisfactory symptom state. Lastly, we observe that women with EOMG display distinct clinical features, in contrast to men with EOMG, who are more like patients with LOMG of both sexes.

A majority of patients in our study (n = 1,035) provided a complete MG-ADL score at inclusion. The score was used to investigate potential determinants of high disease activity and also to estimate PASS. For ePASS, we used the suggested cutoff by Mendoza et al.^9^ and stratified between patients estimated to have achieved a satisfactory symptom state (MG-ADL 0p–2p) and those who had not (MG-ADL ≥3p). Interestingly, a higher frequency of patients diagnosed less than 2 years prior to inclusion reported inadequate ePASS compared to those with longer disease duration. This might be explained by delayed onset of treatment effect, the natural course of the disease, or that patients get used to living with MG.^28^ Taken together, our findings underscore a need to improve treatment algorithms and develop more effective disease-modulatory drugs.

In a multivariate analysis, we observed that sex, obesity, diagnostic delay, and university education were significantly associated to MG-ADL score. In a recently published study investigating predictors for relapse in MG, female sex was identified as a predictive variable for both relapse and reporting lower quality of life.^29,30^ Furthermore, severe disease was associated with low educational attainment and disability pension in a Norwegian study.^31^ The protective effect of a university degree could potentially be associated with increased health care seeking behavior and thereby a more well-treated and stable disease; this was not investigated in the present study. Disease subtype, disease duration, age at study inclusion, or tobacco use were not correlated with MG-ADL score in our study.

A large proportion (40%–85%) of patients with MG present with ocular symptoms but within 2 years approximately 60% of these are reported to have developed generalized symptoms.^3,32,33^ We were not able to estimate time to generalization, but we collected information on when patients experienced peak of disease and with what type of symptoms, and thereafter related it to time of onset. The average time from onset to peak of disease with generalized symptoms (3.0 [SD 5.7] years) is longer than previously reported time to generalization. However, some patients report peak of disease more than 5–10 years after diagnosis, which highlights the importance of continuous follow-up of this condition.

Autoimmune diseases are more common among women in general. In MG, female patients more often develop disease in fertile age, but male patients primarily develop disease at older ages. In our study, we identified striking differences between female and male patients in the EOMG group. Female patients presented with generalized symptoms to a larger extent and developed generalized disease to a much higher degree than both male patients with EOMG and patients with LOMG of both sexes. Female patients with EOMG developed disease at significantly lower age compared to their male counterparts. Female patients with EOMG seem to be a clinically distinct entity whereas male patients with EOMG display characteristics more similar to patients with LOMG. There is no validated environmental risk factor to develop LOMG or EOMG in male patients, whereas sex hormones have been hypothesized to contribute to triggering of EOMG in female patients.^34^ On the basis of shared clinical characteristics, it may be speculated that the pathogenetic mechanism of EOMG in male patients is similar to LOMG, or that the male EOMG group to a larger extent is composed also of individuals with LOMG onset before 50 years of age. Improved biomarkers identifying the 2 subsets could allow for an improved disease stratification in the future, which might be of relevance when selecting therapeutic options such as thymectomy.

The frequency of thymoma in this cohort, 4%, is lower than the 10%–15% reported in previous studies.^35^ This could possibly be a result of self-reported data due to recall bias or that patients have not been adequately informed. However, the frequency of TAMG in our cohort is in line with a recently published study using Swedish mandatory national registries, indicating an increased prevalence of nonthymomatous MG compared to previous cohort-based studies.^36^ Other clinical features are in agreement with previously reported data regarding clinical presentation, age, and sex distributions.^17,18,33^ We performed both internal and external validation to identify potential bias in the cohort. In our validation of reported disease onset, diagnosis, and year of thymectomy, we observed that approximately 5%–10% of answers were not completely overlapping with physician-reported data. However, most answers differed by less than 2 years, indicating a lesser risk of severe recall bias. Because MG is a chronic disease, a larger proportion of patients with EOMG will have had the disease for a longer time and thereby have an increased risk of recall bias. However, in our analysis, disease duration displayed only weak correlation to incorrectly reported data.

This study has some important limitations. First, the reported cohort is estimated to comprise 42% of all patients diagnosed with MG in Sweden and may therefore be subject to selection bias. We were able to invite approximately 60% of the predicted prevalent MG population in Sweden, in relation to an epidemiologic study identifying all individuals receiving an MG ICD code diagnosis in specialized inpatient or outpatient care in 2005–2010. Here, patients were identified through a noncompulsory clinical MG registry, local medical records databases, and the national patient organization. Our method likely results in an enrichment of individuals with severe disease or ongoing immunomodulation requiring more frequent contact with health care, which may overestimate the proportion of patients with unsatisfying symptom states. However, the mean MG-ADL of the cohort is lower than in previous studies, which potentially indicates a more unbiased cohort.^30,37^ Also, diagnostic specificity is likely higher compared to the use of ICD code registries. This notion is supported by the fact that we had to exclude 97 individuals not having MG out of 847 identified in participating hospital registries, after patients contacted us directly or review of medical records of patients from the Stockholm region. Second, as the study is based on patient-reported data, we were not able to obtain serologic information and could thereby only subdivide the cohort based on anamnestic information on presence of thymoma or reported disease onset. Still, in the subgroup of patients identified through the MGreg, prospectively collected physician-reported data showed a high level of agreement with patient-derived information. Third, ePASS was indirectly derived from MG-ADL by calculating the relation between these measures in other reported cohorts. Given the possible existence of differences in cohort structure across studies not accounted for, the calculated outcomes should be interpreted with caution. In addition, MG-ADL is subject to high floor effects and it is possible that patients with MG-associated symptoms exist in the group with 0p.^38^ Lastly, we have not been able to take into account prescribed treatments for the calculated outcomes, such as doses of choline esterase inhibitors or immunomodulatory agents. To provide more precise information on this matter, a study linking the GEMG database with the national prescribed drugs registry is being planned.

We describe characteristics correlating with disease activity in a nationwide prevalence MG cohort covering 42% of the total patient population in Sweden. We show that women and patients with obesity are at higher risk of reporting residual symptoms. Furthermore, almost half of the study population potentially reported an unsatisfactory disease state, highlighting the need for developing improved therapeutic interventions for this condition.

## Glossary

AIC: Akaike information criterion
CI: confidence interval
EOMG: early-onset myasthenia gravis
ePASS: estimated patient acceptable symptom state
GEMG: Genes and Environment in Myasthenia Gravis
ICC: intraclass coefficient
ICD: International Classification of Disease
LOMG: late-onset myasthenia gravis
MG: myasthenia gravis
MG-ADL: myasthenia gravis activities of daily living
MGreg: national clinical MG registry
OR: odds ratio
PASS: patient acceptable symptom state
PROM: patient-reported outcome measure
TAMG: thymoma-associated myasthenia gravis
